# Hydro-sedimentary provenance analyses in the Weiße Elster catchment (Central Germany): The basic dataset

**DOI:** 10.1016/j.dib.2021.107719

**Published:** 2021-12-16

**Authors:** Helen Ballasus, Hans von Suchodoletz, Birgit Schneider, Hermann Grün, Anna Heller, Marie-Sophie Kind, Bennet Wroblewski, Stefan Wurlitzer, Christoph Zielhofer

**Affiliations:** Chair of Physical Geography, Institute of Geography, Leipzig University, Johannisallee 19a, D-04103 Leipzig, Germany

**Keywords:** Provenance analysis, Stationary XRF data, Grain size data, Floodplain, Catchment-scale, Hydro-sedimentary dynamics, Neolithic, Central Germany

## Abstract

This manuscript documents geological master data and X-ray fluorescence (XRF) data of a standardized 8*8 km sampling grid of the entire Weiße Elster catchment in Central Germany. Further, the manuscript documents XRF data of a refined 4*4 km sampling grid in the proximity of Salsitz floodplain transect as well as grain size data and XRF data of Salsitz SC40 core that was recovered from the Weiße Elster floodplain. The data provide opportunities for hydro-sedimentary provenance analyses as presented in the corresponding research article by Ballasus et al. [1].

## Specifications Table

**Table:** 

Subject	Earth and Planetary Sciences – Earth-Surface Processes, Stratigraphy
Specific subject area	Hydro-sedimentary provenance analysis, catchment-scale approach, XRF analysis, Fluvial Geomorphology, Holocene, overbank silt-clay deposition
Type of data	Tables, Figures
How data were acquired	For XRF sediment provenance analysis in the Weiße Elster catchment of Central Germany sediment bulk material of recent streambeds from sub-catchments were sampled within a systematic mesoscale framework. A grid of 8*8 km cells was arranged over the entire Weiße Elster catchment. Thus, the extracted sediments contain the geochemical signal of the respective sub-catchment. The sub-catchments samples were classified according to their geological features. Based on the grid, a map was created in which the primarily represented geological unit were assigned as a raster-cell parameter. Further, a core was recovered from the Weiße Elster floodplain and a total number of 52 geochemical samples was obtained from this SC40 core. Subsequently, stationary XRF spectrometry (Spectro Xepos energy dispersive XRF spectrometer) was applied for analysing elemental composition of bank and streambed samples from the Weiße Elster sub-basins (111 grids) and SC40 core samples. In addition, grain size analysis from SC40 core samples were conducted using dry-sieving technique for the sand fraction and a SediGraph III 5120 (Micromeritics) for the silt and clay fraction. GRADISTAT v9.1 for the calculation of D50 (median of grain size), D75/D25 and D75-D25 (interquartile range of grain size distribution) was used.
Data format	Raw Analyzed
Parameters for data collection	Grain size data were clustered (2000–630 µm: coarse sand, 630–200 µm: medium sand, 200–125 µm: fine sand, 125–63 µm: finest sand, 63–20 µm: coarse silt, 20–6.3 µm: medium silt, 6.3–2.0 µm: fine silt, 2.0–0.6 µm: coarse clay, 0.6–0.2 µm: medium clay and <0.2 µm: fine clay). XRF data are in mg/kg.
Description of data collection	Sediment bulk material were sampled from recent streambeds of Weiße Elster sub-catchments with a spade. At few sites with complex fluvial stratigraphies, duplicate samples were taken and then mean values were calculated subsequently. Within the Weiße Elster floodplain at Salsitz SC40 position two parallel overlapping core runs were recovered using vibra-coring.
Data source location	Weiße Elster catchment Central Germany Median Latitude: 50.899010 Median Longitude: 12.252178 South-bound Latitude: 50.245682 West-bound Longitude: 11.813339 North-bound Latitude: 51.530911 East-bound Longitude: 12.680526
Data accessibility	Repository name: Pangaea Data Publisher Ballasus, H., Zielhofer, C., von Suchodoletz, H., Schneider, B., 2021. Grain size data sheet: Weiße Elster overbank silt-clay deposition (SC40 core, transect Salsitz). PANGAEA https://doi.org/10.1594/PANGAEA.937901 Repository name: Pangaea Data Publisher Ballasus, H., Zielhofer, C., 2021. Master data sheet: Weiße Elster catchment sampling (fluvial sediments) within an 8*8 km grid as sampling template. PANGAEA https://doi.org/10.1594/PANGAEA.937898 (DOI registration in progress) Repository name: Pangaea Data Publisher Ballasus, H., Zielhofer, C., Schneider, B., 2021. XRF element data of fluvial deposits from the Weiße Elster catchment. PANGAEA https://doi.org/10.1594/PANGAEA.937926 (DOI registration in progress) Repository name: Pangaea Data Publisher Ballasus, H., Zielhofer, C., Schneider, B., von Suchodoletz, H., 2021. XRF data sheet: overbank silt-clay deposition sampling (4*4 km grid, Weiße Elster catchment, Salsitz-Trebnitz section). PANGAEA https://doi.org/10.1594/PANGAEA.937937 (DOI registration in progress) Repository name: Pangaea Data Publisher
	Zielhofer, C., Ballasus, H., Schneider, B., von Suchodoletz, H., 2021. XRF data sheet: Weiße Elster overbank silt-clay deposition (SC40 core, Salsitz transect). PANGAEA, https://doi.org/10.1594/PANGAEA.937939 (DOI registration in progress)
Related research article	Ballasus, H., Schneider, B., von Suchodoletz, H., Miera, J., Werban, U., Fütterer, P., Werther, L., Ettel, P., Veit, U., Zielhofer, C., 2022. Overbank silt-clay deposition and intensive Neolithic land-use in a Central European catchment – coupled or decoupled? Science of the Total Environment 806: 150858, https://doi.org/10.1016/j.scitotenv.2021.150858

## Value of the Data

• The presented data provide information about the elemental composition and the geological background of fluvial deposits in the Weiße Elster catchment.
• Geoscientists and geosciences students can use the data for applying and improving hydro-sedimentary provenance analyses.
• The data can be used with other provenance parameters such as strontium (Sr), neodymium (Nd) and lead (Pb) isotopes providing further insights and/or developments of provenance models.

## 1 Data Description

(a) Master data of the standardized 8*8 km sampling grid (Weiße Elster catchment)

**Fig. 1:**
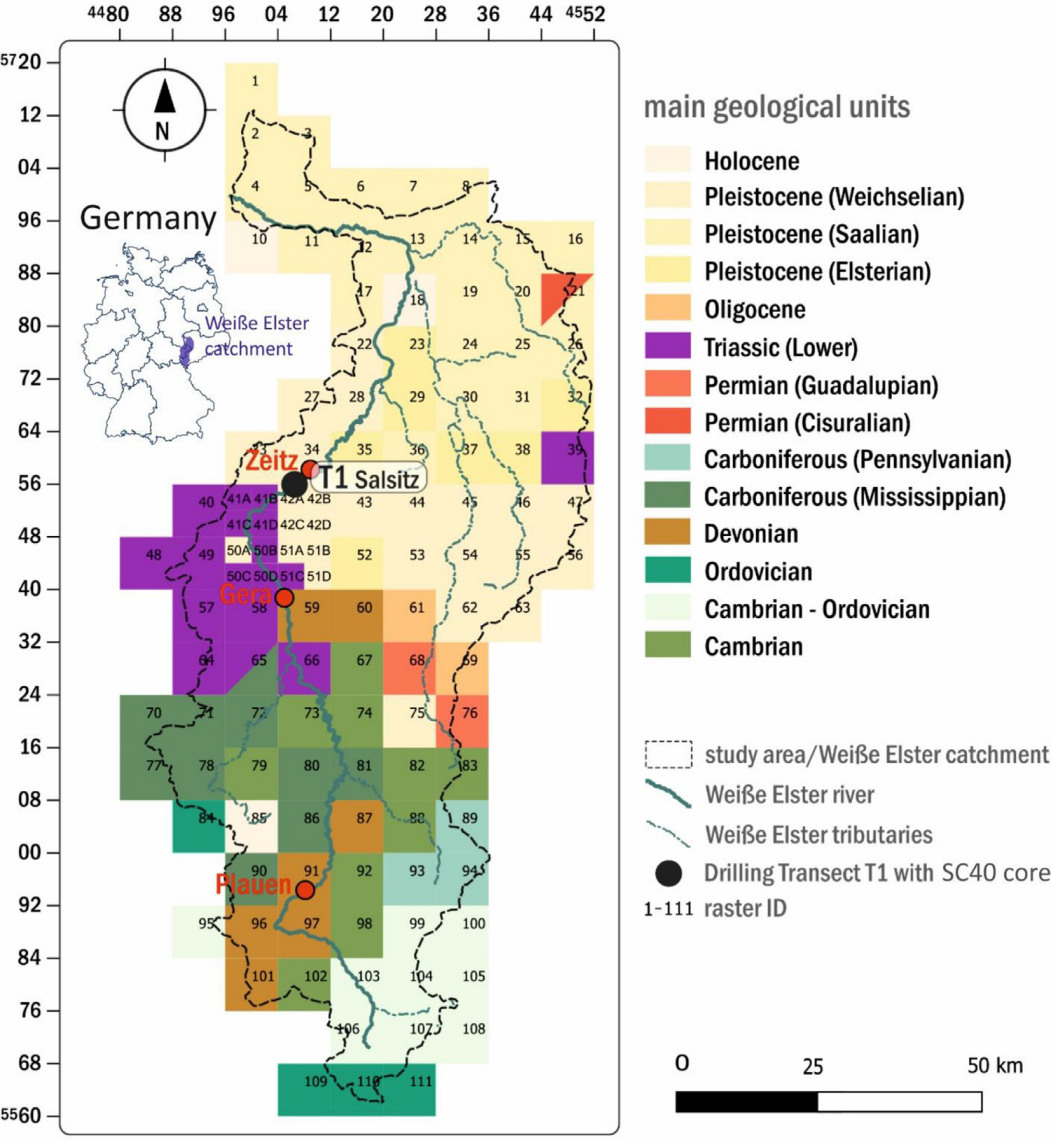
Sampling grids classified by their dominant sub-basin's geology. Grids divided into two colours mark sub-catchments, which represent two dominant geological areas of equal size. Colour coding corresponds with Cohen et al. [3].

The master data of the standardized 8*8 km sampling grid of recent fluvial sediments across the Weiße Elster catchment (Fig. 1) are provided in the data file “Master data sheet: Weiße Elster catchment sampling (fluvial sediments) within an 8*8 km grid as sampling template” [2]. Table 1 provides explanations of the parameters for each column. The sample/raster ID "WE 009*" was not sampled. The corresponding grid cell is located at the edge of the Weiße Elster catchment and no suitable tributary for the extraction of alluvial sediment could be found.

(b) XRF data of the standardized 8*8 km sampling grid (Weiße Elster catchment)

**Table 1:** Weiße Elster catchment: master data file description.

Column	Parameter	Description
1 2	Event label Sample code	Code of the sampling site Identification number of sediment sample, WE = Weiße Elster; [character, unitless]
3 4	Latitude Longitude	Latitude in decimal degree Longitude in decimal degree
5 6	Elevation Sampling date	Height above sea level; [m] Date of sampling; [YYYY-MM-DD]
7	Location	Name of the tributary; [character, unitless]
8	Dominant geology	Dominant chronostratigraphical unit within the sampled sub-catchment, according to the international chronostratigraphical chart (Cohen et al. [3]; [character, unitless]
9	German soil texture class	German soil texture class: according to Ad-hoc-AG Boden [4], estimated by finger test; [character, unitless]
10	FAO soil texture class	FAO soil texture class deduced from the German soil texture classes (H) by Helen Ballasus [character, unitless]
11	Munsell colour code	Munsell colour code from alignment with Munsell soil colour charts [character, unitless]
12	Munsell colour	Munsell colour from alignment with Munsell soil colour charts; [character, unitless]
13	Water course section	water course section: ur = upper reaches, mr = middle reaches, lr = lower reaches [character, unitless]
14	Tributary catchment size	Catchment size of sampled tributary (of the Weiße Elster river) upstream of the sampling point, estimated by using maps; [km^2^]

The XRF element data of the standardized 8*8 km sampling grid of recent fluvial sediments across the Weiße Elster catchment (Fig. 1) are provided in the data file “XRF element data of fluvial deposits from the Weiße Elster catchment” [5]. Table 2 provides explanations of the parameters for each column. The sample ID "WE 009" does not exist. Duplicate samples from the same position are marked with an “a” and “b”.

**Table 2:** Weiße Elster catchment: XRF element data file description (8*8 km grid).

Column	Parameter	Description
1	Event label	Code of the sampling site
2 3	Latitude Longitude	Latitude in decimal degree Longitude in decimal degree
4	Sample ID	Designation of sediment sample, WE = Weiße Elster; [character, unitless]
5-104	Total element contents and absolute errors	Total contents of the elements (range from sodium to uranium) [mg/kg] with indication of the absolute error for each element listed as `ELEMENT e [±]'
105	Total [%]	Sum of all measurable element contents [%]

Comments within the XRF element data file (8*8 km grid) indicate the measurement reproducibility: *“Very good”* indicates very good reproducibility with highly reliable data. “Very good**” indicates very good reproducibility with highly reliable data but individual values are not secured, since concentrations are based on measurements with < 1000 pulses. Not secured samples are Cl contents in columns 17/18 with values < 2.0 and the Th content of sample WE 020 in columns 101/102.

*“Moderate”* indicates that values can be used in terms of proportions within the data set. “Moderate*” indicates that values can be used in terms of proportions within this data set but individual values are secured, since concentrations are based on measurements with >1000 pulses. Secured samples are Se contents in columns 47/48 with sample ID WE 004, WE 008, WE 010, WE 016, WE 018, WE 019, WE 022, WE024, WE025, WE 029, WE 030, WE 036, WE 059, WE 060, WE 075, WE 075b, WE 082, WE 082b, WE 087 and WE 111. “Moderate* **” indicates that values can be used in terms of proportions within this data set but individual values are secured, since concentrations are based on measurements with >1000 pulses. Secured samples are Co contents in columns 33/34 with sample ID WE 010, WE 015, WE 022-WE 025, WE 029, WE 030, WE 032, WE 049, Nb contents in columns 59/60 with sample ID WE 001, WE 003, WE 004, WE 006, WE 008, WE 010, WE 013, WE 014, WE 016-WE 019, WE 021-WE 023, WE 025-WE 032, WE 034, WE 036-WE 039, WE 041-WE 047, WE 050-WE 054 and U contents in columns 103/104 with sample ID WE 059, WE 060, WE 075, WE 075b, WE 083 and WE 089. Individual values are not secured, since concentrations are based on measurements with < 1000 pulses. Not secured samples are Co contents in columns 33/34 with sample ID WE 033, WE 049, Nb contents in column 59/60 with sample ID WE 057, WE 062 and WE 062b and U contents in columns 103/104 with sample ID WE 002, WE 005, WE 011-WE 015, WE 020 and WE 033.

*“Poor”* indicates that data should be treated with caution, since the values were measured close to the detection limit. “Poor*” indicates that data should be treated with caution, since the values were measured close to the detection limit but individual values are secured, since concentrations are based on measurements with >1000 pulses. Secured samples are Mo contents in columns 61/62 with values >10.4, Cd contents in columns 65/66 with sample ID WE 035, Sn contents in columns 67/68 with sample ID WE 010, WE 012, WE 016, WE 025, WE 030, WE 035, WE 046, WE 056, WE 056b, WE 066, WE 069, WE 069b, WE 075, WE 089, WE 089b, WE 093, WE 093b, Sb contents in columns 69/70 with sample ID WE 059, WE 069, WE 069b, WE 087, WE 094, WE 094b, Te contents in columns 71/72 with sample ID WE 016, WE 026, I contents in columns 73/74 with sample ID WE 016, WE 022, WE 030, WE 031, WE 045, WE 052, Cs content in columns 75/76 with sample ID WE 016, WE 048 and Hg content in columns 93/94 with sample ID WE 010.

(c) XRF data of the refined 4*4 km sampling grid (proximity to Salsitz transect)

**Fig. 2:**
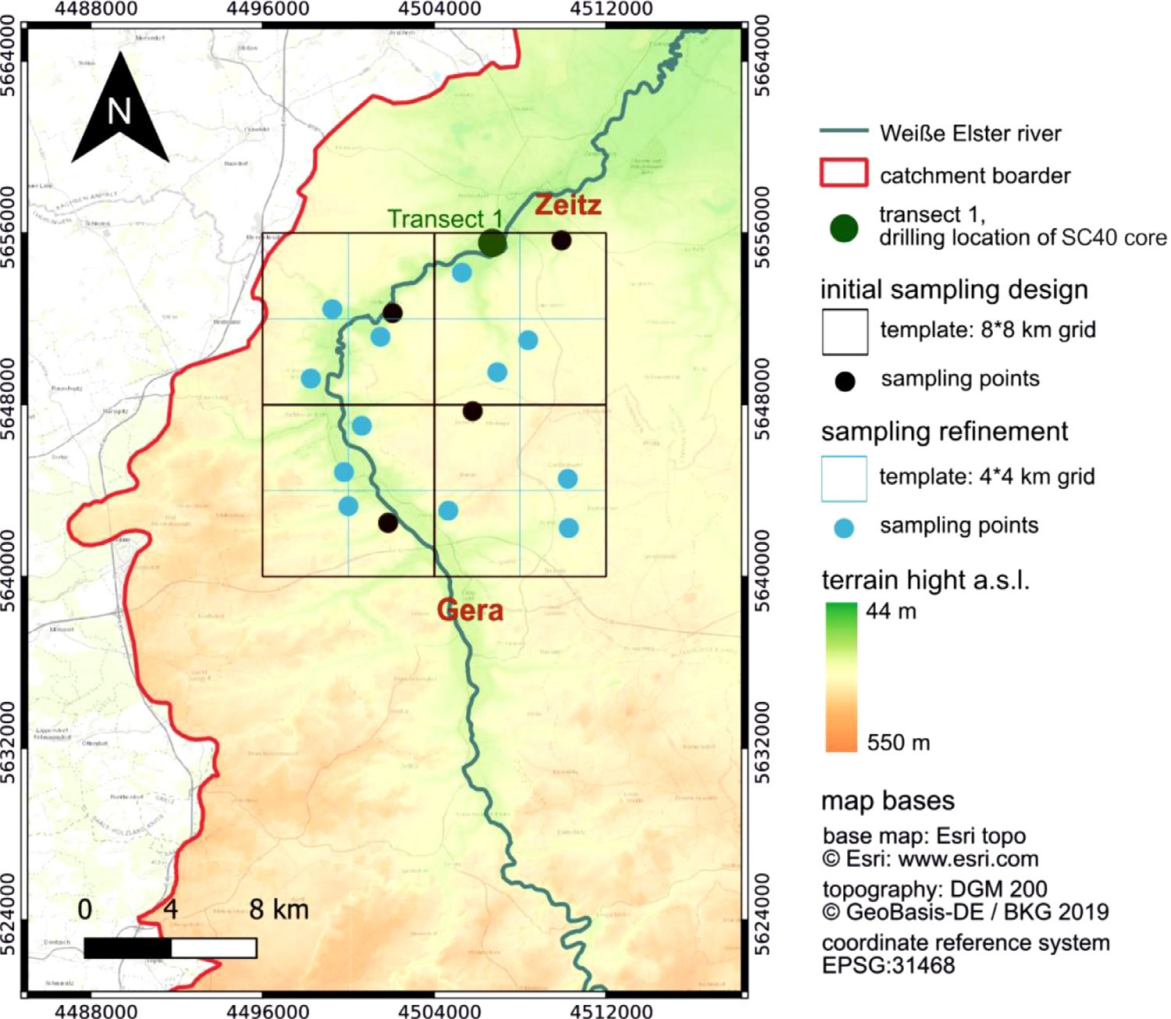
Small-scale sampling within a 4*4 km sampling grid in the direct proximity to the Salsitz transect.

The XRF element data of the refined 4*4 km sampling grid of recent fluvial sediments in the direct proximity to the Salsitz transect (Fig. 2) are provided in the data file “XRF data sheet: 4*4 km grid, Weiße Elster catchment, Salsitz-Trebnitz section)” [6]. Table 3 provides explanations of the parameters for each column. The data set has to be understood as a supplement or densification of the 8*8 km sampling grid of the Weiße Elster river catchment (Ballasus et al. [5]).

**Table 3:** Weiße Elster catchment: XRF element data file description (refined 4*4 km grid).

Column	Parameter	Description
1	Event label	Code of the sampling site
2	Sample ID	Designation of sediment sample, WE = Weiße Elster; [character, unitless]
3	Latitude	Latitude in decimal degree
4	Longitude	Longitude in decimal degree
5-104	Total element contents and absolute errors	Total contents of the elements (range from sodium to uranium) [mg/kg] with indication of the absolute error for each element listed as `ELEMENT e [±]'
105	Total [%]	Sum of all measurable element contents [%]
		

Comments within the refined XRF element data file (4*4 km grid) indicate the measurement reproducibility: *“Very good”* indicates very good reproducibility with highly reliable data. “Very good**” indicates very good reproducibility with highly reliable data but individual values are not secured, since concentrations are based on measurements with < 1000 pulses. A not secured sample is W content in columns 91/92 with sample ID WE_50C.

*“Moderate”* indicates that values can be used in terms of proportions within the data set. “Moderate*” indicates that values can be used in terms of proportions within this data set but individual values are secured, since concentrations are based on measurements with >1000 pulses. Secured samples are Se contents in columns 47/48 with sample ID WE_41A, WE_50C, WE_51B and WE_51D. “Moderate**” indicates that values can be used in terms of proportions within this data set but individual values are not secured, since concentrations are based on measurements with <1000 pulses. Not secured samples are Nb contents in columns 59/60 with sample ID WE_41C and WE_50C.

*“Poor”* indicates that data should be treated with caution, since the values were measured close to the detection limit.

(d) *Field description of SC40 core*

**Fig. 3:**
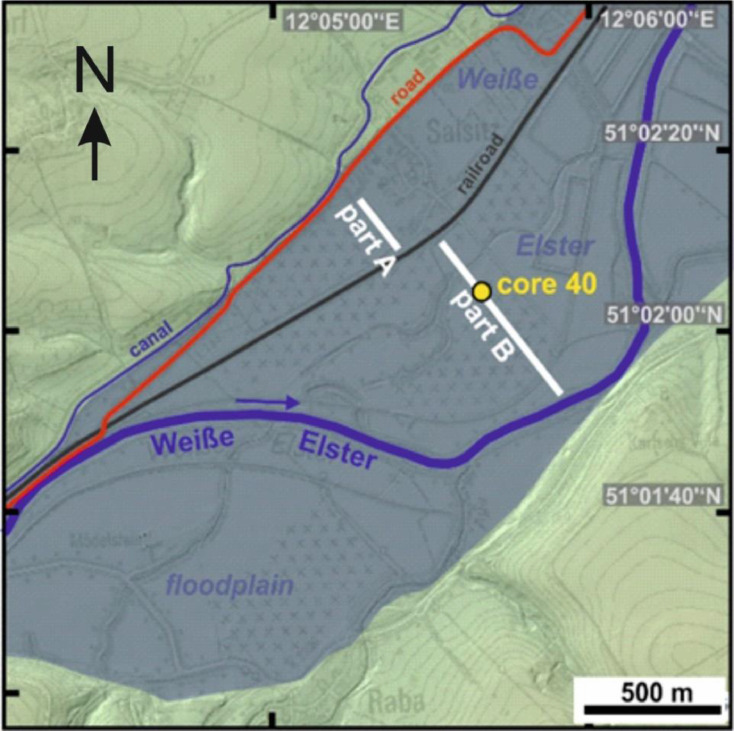
Location of the Salsitz transect with SC40 core.

Salsitz SC40 core is located in the Weiße Elster floodplain (Fig. 1). The stratigraphy of SC40 core (Fig. 3, Latitude: 51.034520, Longitude: 12.093900) with a total depth of 400 cm reveals a gravelly braidplain/channel deposit at the base. This is overlain by a sandy bedform that grades upwards into an overbank silt-clay deposition (named “lower overbank silt-clay deposition”) that is overprinted by an intensively developed paleosol (paleosol S-PS-3). This paleosol is overlain by three packages of overbank silt-clay deposition (named “upper overbank silt-clay deposition”), which are separated from each other by two paleosols (paleosols S-PS-2 and S-PS-1). A recent plow horizon (Ap) is found at the floodplain surface (Table 4).

(e) *Grain size data of Salsitz SC40 core (Weiße Elster floodplain)*

**Table 4:** Field description of core SC40 from drilling transect Zeitz.

Depth (cm)	Substrate/soil horizon	Munsell colour	Description
0-45	Overbank silt-clay deposition with plow horizon (Ap)	dark reddish brown (5 YR 3/4)	Clay loam (CL), singular gravels up to 0.5 cm

45-79	Overbank silt-clay deposition	dark yellowish brown (10 YR 3/6)	Clay loam (CL) with fining upwards, singular gravels up to 0.5 cm

79-84	Overbank silt-clay deposition with **paleosol S-PS-1**	dark yellowish brown (10 YR 4/4)	Clay loam (CL), greyish/brownish mottling, singular Mn-concretions up to 4 mm

84-119	Overbank silt-clay deposition	dark yellowish brown (10 YR 4/6 – 10 YR 4/4)	Silty clay loam (SiCL), slight greyish/brownish mottling

119-124	Overbank silt-clay deposition with **paleosol S-PS-2**	dark yellowish brown (10 YR 4/4)	Clay loam (CL), some Fe-concretions and Mn-concretions up to 4 mm

124-169	Overbank silt-clay deposition	yellowish brown (10 YR 5/4)	Silty clay loam (SiCL) with coarsening upwards, some Mn-concretions, 135 – 160 cm concentration of charcoal pieces

169-189	Overbank silt-clay deposition with **paleosol S-PS-3**	brown (7.5 YR 4/4)	Clay loam (CL), partly black mottling, large Mn-concretions, charcoal in upper part

189-280	Overbank silt-clay deposition	brown - strong brown (7.5 YR 4/4 - 7.5 YR 4/6)	Clay loam (CL) (250-260 cm lens of Sandy clay loam (SCL)), large bleached cracks/root channels

280-300	Sandy bedform	bluish grey (10 PB 6/1)	Middle sand with fining upwards

300-400	Braidplain/Channel deposit		gravel up to 5 cm in matrix of middle to coarse sand

Salsitz SC40 core is located in the Weiße Elster floodplain (Fig. 1). The grain size data of Salsitz SC40 core (Fig. 3) are provided in the data file “Grain size data sheet: Weiße Elster overbank silt-clay deposition (SC40 core, transect Salsitz)” [7]. The total drilling depth of SC40 core is 275 cm. Table 5 provides explanations of the parameters for each column.

(f) *XRF data of Salsitz SC40 core (Weiße Elster floodplain)*

**Table 5:** Salsitz SC40 core: grain size data file description.

Column	Parameter	Description
1	sample code	designation of sediment sample, WE = Weiße Elster, SC = Salsitz core; [character, unitless]
2	sampling depth (top/min)	minimum extraction depth of sediment sample (measured from surface); [m]
3	sampling depth (bottom/max)	maximum extraction depth of sediment sample (measured from surface); [m]
4	sampling depth (average)	average extraction depth of sediment sample (measured from surface); [m]
5-14	Grain size classes	Grain size classes according to DIN 4022 (German Institute for Standardisation); [%]
15	D50	Median of grain size [µm], 50% of grains are smaller
16	D75/D25	D75 (75% of grains are smaller) divided by D25 (25% of grains are smaller)
17	D75-D25	Interquartile range of grain size distribution [µm], D25 (25% of grains are smaller) substracted from D75 (75% of grains are smaller)

The XRF element data of Salsitz SC40 core (Fig. 3) are provided in the data file “XRF data sheet: Weiße Elster overbank silt-clay deposition (SC40 core, Salsitz transect)” [8]. The total drilling depth of SC40 core is 275 cm. The authors conducted stationary XRF measurements of 52 samples. Table 6 provides explanations of the parameters for each column.

**Table 6:** Salsitz SC40 core: XRF element data file description.

Column	Parameter	Description
1	sample code	designation of sediment sample, WE = Weiße Elster, SC = Salsitz core; [character, unitless]
2	sampling depth (top/min)	minimum extraction depth of sediment sample (measured from surface); [m]
3	sampling depth (bottom/max)	maximum extraction depth of sediment sample (measured from surface); [m]
4	sampling depth (average)	average extraction depth of sediment sample (measured from surface); [m]
5-104	Total element contents and absolute errors	Total contents of the elements (range from sodium to uranium) [mg/kg] with indication of the absolute error for each element listed as `ELEMENT e [±]'
105	Total [%]	Sum of all measurable element contents [%]

Comments within the XRF element data file indicate the measurement reproducibility: *“Very good”* indicates very good reproducibility with highly reliable data. “Very good**” indicates very good reproducibility with highly reliable data but individual values are not secured, since concentrations are based on measurements with < 1000 pulses. Not secured samples are Br contents in columns 49/50 with sample ID WE_SC40-50 to WE_SC40-52.

*“Moderate”* indicates that values can be used in terms of proportions within the data set. “Moderate*” indicates that values can be used in terms of proportions within this data set but individual values are secured, since concentrations are based on measurements with >1000 pulses. Secured samples are Cl contents in columns 17/18 with sample ID WE_SC40-1 to WE_SC40-8, WE_SC40-11 to WE_SC40-13 and WE_SC40-22 to WE_SC40-23.

*“Poor”* indicates that data should be treated with caution, since the values were measured close to the detection limit.

## 2 Experimental Design, Materials and Methods

(A) *Sediment source sampling in the Weiße Elster catchment*

In autumn 2019, sediment bulk material of recent streambeds was sampled from sub-catchments within a systematic mesoscale framework (see rasters 1 to 111 in Fig. 1). In this context, a grid of 8*8 km cells was arranged over the entire Weiße Elster catchment, yielding a total of 111 raster cells. In each raster cell, recent streambed deposits from Weiße Elster tributaries were sampled with a spade with a maximum depth of 20 cm. Thus, the extracted sediments contain the geochemical signal of the respective sub-catchment. At few sites (raster cells 56, 62, 69, 75, 82, 85, 93, 94 in Fig. 1) with complex fluvial stratigraphies, duplicate samples were taken and then mean values were calculated from the subsequent XRF analysis. Complex fluvial stratigraphies exist when streambed deposits were not accessible and adjacent sampling sites had to be used that were just outside the recent streambed.

Since the 8*8 km sampling approach might not necessarily capture geochemical features of proximal hillslope sediments at Salsitz SC40 core and in order to verify the reliability of the sampling approach, the grid close to the Salsitz floodplain section was further refined (4*4 km grids, Fig. 2).

The 111 sub-catchments samples were classified according to their geological features. Based on the grid, a map was created in which the primarily represented geological units were assigned (largest area in the sub-basin) as a raster-cell parameter (Fig. 1).

(B) *Weiße Elster floodplain: recovery and sampling of SC40 core*

Within the Weiße Elster floodplain at Salsitz SC40 position (Fig. 3) two parallel overlapping cores were recovered and sampled. A total number of 52 geochemical samples was obtained from Salsitz SC40 core by averaging sediment material over a length of ca. 5 cm.

(C) *Weiße Elster catchment and Salsitz SC40 core: XRF-based element analyses*

A stationary XRF spectrometry was used for analysing elemental composition of bank and streambed samples from the Weiße Elster sub-basins (111 grids) and SC40 core samples. For XRF-sample preparation freeze-dried core and catchment sediments (8 g) were seaved (2mm) to discard the gravel fraction and large organic matter. Further homogenization was undertaken by grinding the samples with a vibratory Retsch mill MM 200. Uniform pellets were created by pressing the powdered samples with a carbon-based binding agent in a Vaneox press at 20 t for 2 min [9]. Elemental analysis was conducted in a He atmosphere using a Spectro Xepos energy dispersive XRF spectrometer.

(A) *Salsitz SC40 core: grain size analysis of the floodplain deposits*

The 2 mm sieved samples (10 g) were left in 50 ml 35-% hydrogen peroxide (H_2_O_2_) overnight and heated during the next day to remove organic matter. Subsequently, the samples were dispersed using a 10 ml 0.4 N sodium pyrophosphate solution (Na_4_P_2_O_7_) and ultrasonic treatment for 45 min [10]. The grain size analysis of the sand fraction was conducted by means of the dry-sieving technique (2000–630 µm: coarse sand, 630–200 µm: medium sand, 200–125 µm: fine sand, 125–63 µm: finest sand). The finer fractions (63–20 µm: coarse silt, 20–6.3 µm: medium silt, 6.3–2.0 µm: fine silt, 2.0–0.6 µm: coarse clay, 0.6–0.2 µm: medium clay and <0.2 µm: fine clay) were measured by X-ray granulometry (XRG) using a SediGraph III 5120 (Micromeritics). GRADISTAT v9.1 [11] was used for the calculation of D50 (median of grain size), D75/D25 and D75-D25 (interquartile range of grain size distribution).

## Ethics Statement

The authors declare that this submission follows the ethical requirements for publication in Data in Brief.

## CRediT authorship contribution statement

**Helen Ballasus:** Conceptualization, Methodology, Software, Validation, Formal analysis, Investigation, Data curation, Writing – original draft, Visualization, Project administration. **Hans von Suchodoletz:** Investigation, Resources, Writing – review & editing, Funding acquisition. **Birgit Schneider:** Investigation, Data curation. **Hermann Grün:** . **Anna Heller:** . **Marie-Sophie Kind:** Formal analysis, Investigation. **Bennet Wroblewski:** . **Stefan Wurlitzer:** . **Christoph Zielhofer:** Conceptualization, Methodology, Validation, Formal analysis, Resources, Writing – original draft, Writing – review & editing, Supervision, Funding acquisition.

## Declaration of Competing Interest

The authors declare that they have no known competing financial interests or personal relationships, which have or could be perceived to have influenced the work reported in this article.
